# Knowledge and Attitudes of Parents towards Childhood Hearing Loss and Pediatric Hearing Services in Sharjah, United Arab Emirates

**DOI:** 10.3390/ijerph18126188

**Published:** 2021-06-08

**Authors:** Muhammed Ayas, Hakam Yaseen

**Affiliations:** 1 Audiology Unit, University Hospital Sharjah, Sharjah 72772, United Arab Emirates; 2 Department of Clinical Sciences, College of Medicine, University of Sharjah, Sharjah 72772, United Arab Emirates; hakam.yaseen@uhs.ae; 3 Neonatology and Paediatrics, University Hospital Sharjah, Sharjah 72772, United Arab Emirates

**Keywords:** parental attitudes, parental knowledge, hearing loss, pediatric audiology, awareness

## Abstract

The successful implementation of pediatric audiology services depends on parental engagement and support. It is essential to analyze the gaps in knowledge level and attitude of the parents in United Arab Emirates (UAE), towards pediatric hearing loss and pediatric audiology services. The present study aimed to assess the knowledge and attitude of parents in Sharjah, UAE.A cross-sectional survey was administered to 295 parents in a tertiary care hospital setting. The self-reported questionnaire consisted of 26 items. 34.2% of the parents ascertaining good knowledge and 65.8% reported poor knowledge regarding the various factors related to the childhood hearing loss. Further, 86.2% of parents reported positive attitudes regarding accessing pediatric audiology services. A significant association was found between age groups, educational status, and knowledge levels. The study highlights the poor knowledge demonstrated by parents in the UAE regarding hearing loss and its associated risk factors. Findings outline the critical need in the region to enhance parental awareness. More health promotion activities and community outreach campaigns are necessary to increase the uptake of pediatric audiology services in the region.

## 1. Introduction

The development and implementation of successful pediatric audiological services relies on active participation and support from parents within the community [1]. Such dependencies highlight the need for a positive attitude and high levels of awareness among parents towards hearing loss. These factors are critical in developing a sustainable, need-based comprehensive hearing care program for children with hearing loss [2,3]. Recent data on hearing loss suggest that globally 20% of people have hearing loss. By 2050, it is projected that 2.45 billion people will have some amount of hearing loss [4], including a vast percentage of the pediatric population. In the Middle East, there are limited data available on the true prevalence of pediatric hearing loss. A recent study reports that the overall prevalence of pediatric hearing loss is close to 3.7 in 1000 [5]. 

Here is a general negative attitude reported among parents towards hearing screening and hearing loss [6,7]. Usually, childhood hearing loss is detected through suspicion raised by family members [8]. However, where such detection may lack, due to less close family members, the identification of hearing loss may be delayed. In turn, this may have detrimental long-term implications on the child’s overall development.

Studies have been conducted to assess the knowledge and attitude of parents towards hearing loss in many countries. In the middle east, particularly in Saudi Arabia, a recent study revealed that less than 50% of parents have adequate knowledge about hearing loss, whereas approximately 93% of parents showed a positive attitude towards audiology related services [9]. However, contradictory findings were reported in another study—60% of the parents and family members reported a fair degree of knowledge about topics such as high fever, ear discharge, and noise exposure [10]. Additionally, fathers exhibited a comparatively higher degree of knowledge than mothers regarding breast-feeding (*p* = 0.031), Otitis Media (OM) (*p* = 0.038), and noise exposure (*p* = 0.007) [11].

Studies report that parents showed a positive attitude and favored seeking audiological services [9]. Most parents agreed to have newborn hearing screening done at birth. However, a concern remained among parents regarding the use of hearing aids for their child [12]. Many studies have reported poor community awareness regarding infant hearing loss [9,13,14]. However, with the current advances in technology and social media, there may be grounds for raising awareness within the community. Therefore, it is vital for us to assess the parental knowledge and attitude towards childhood hearing loss and associated services available for children. 

To the best of our knowledge, there are limited or no published data available on parental knowledge and attitudes towards hearing loss in the United Arab Emirates (UAE). Thus, the present study attempts to explore the parental knowledge and attitude towards childhood hearing loss and pediatric hearing services in Sharjah, UAE, through a cross-sectional survey of the parents.

## 2. Materials and Methods

### 2.1. Ethical Approval

The study was in accordance with the Code of Ethics of the World Medical Association (Declaration of Helsinki). Ethical approval was obtained for the current study from the Institutional Ethics and Research Committee of University Hospital Sharjah (UHS-HERC-041-01092020).

### 2.2. Participants 

A cross-sectional survey study was conducted. The study was carried out from September 2020 to January 2021 at a tertiary care university hospital in Sharjah, UAE. The survey was administered to 295 parents of children without an hearing impairment diagnosis, who visited the well-baby clinic and an audiology clinic for a pediatric hearing assessment. We targeted both fathers and mothers of the newborns, regardless of them being first-time or subsequent parents. Due to the cultural sensitivities in the region, we targeted both parents to measure and compare their responses. To be included in the study, parents should be aged 20 years and above. Parents with below-elementary level of education and who could not comprehend the language were excluded from the study. Both parents were given a thorough explanation regarding the purpose of the study. Written consent was obtained before the paper-based questionnaire was handed and answeredanonymously by the parents

### 2.3. Questionnaire

The questionnaire consisted of 26 items (Table 1). Twenty questions evaluated the knowledge pertaining to the causes and risk factors of sensorineural hearing loss (SNHL), OM, and identification of hearing loss and intervention. The remaining six questions targeted the attitude of parents towards seeking timely audiological services. The questions were phrased after reviewing the existing studies on the knowledge and attitudes of parents towards infant and childhood hearing loss [9,11]. Certain risk factors were adopted from the Joint Committee on Infant Hearing (JCIH) positional statement [15]. Due permission was obtained from the authors who primarily developed the questionnaire before the study was initiated [11]. The final version of the questionnaire was translated to Arabic and back translated into English by four experienced professional language translators, proficient in both languages.

After the translation, the Arabic version of the questionnaire was shared with two audiologists, two otolaryngologists, and two neonatologists, who were asked to assess the questionnaire and rule out any ambiguities in the regional context. Their suggestions were incorporated, and a pilot study was conducted on 20 parents (10 fathers, 10 mothers) to ensure the clarity of the questions and to estimate the duration needed to complete the questionnaire. The pilot study questions were to be rated on a three-point rating scale: yes, no, or unsure. Parental demographic information, including age and level of education, was also collected.

### 2.4. Statistical Analysis

The data were entered and analyzed using the Statistical Package for Social Sciences (SPSS V.21, IBM, Armonk, NY, USA). Descriptive statistics were utilized to describe the frequency of the responses and analyzed in percentages. Twenty questions evaluated knowledge levels and six questions assessed attitudes. For ease of interpretation, responses were further coded as “Yes” = 1, “No” and “Unsure” = 0. Knowledge and attitude levels were computed based on a total of 20 points and 6 points, respectively. Knowledge responses were further grouped into: “poor knowledge” = 0–10 points and “good knowledge” = 11–20 points. Attitude was scored based on the range: “negative” for 0–3 points and “positive” for 4–6 points. Furthermore, the chi-square test was utilized to measure the association between socio-demographic details and the knowledge and attitude responses. The significant level was kept at a *p*-value of 0.05. The internal consistency of the questionnaire was assessed via Cronbach’s alpha coefficient.

## 3. Results

A total of 295 parents participated in the study. The overall response rate was 91.2% (269). Ninety-four (34.9%) fathers and 175 (65.1%) mothers completed the questionnaire. The mean age of the participants was 30.7 years (standard deviation: 5.5) and ranged from 21–49 years. One-hundred and eighty-eight (69.9%) parents were university graduates and 81(30.1%) were educated at secondary levels or below.

### 3.1. Knowledge and Attitudes of the Parents

Table 1 summarizes the knowledge and attitudes of the parents towards childhood hearing loss. Both fathers and mothers indicate a high level of knowledge, in response to the question on “consanguineous marriage and hearing loss” (57.5% and 42.4%, respectively). They also showed a high degree of acknowledgment to their information on “babies can be born with hearing loss” (48.2% and 44.6%, respectively).

Questions addressing maternal rubella (5.5%, 11.1%) and low birth weight as risk factors (14.5%, 7.3%), showed extremely poor awareness among fathers and mothers, respectively. In addition, their knowledge related to risk factors, such as OM and conductive hearing loss, was rated high. Both fathers (55.1%) and mothers (47.2%) reported that were aware that “Ear discharge and OM cause hearing loss.”

Regarding identification and intervention, both fathers (77.1%) and mothers (56.5%) claimed that were fully aware that “children with hearing loss can attend schools.” Interestingly, they showed a great degree of understanding regarding “speech and language problems can be a sign of hearing loss” (69.5% and 61.5%, respectively). Moreover, both fathers and mothers showed positive and agreeable attitudes towards availing childhood audiology services.

### 3.2. Overall Parental Level of Knowledge and Attitudes

Overall responses to parental knowledge and attitudes are illustrated in Table 2. Of the 269 parents, 92(34.2%) indicated having good knowledge about the various factors associated with childhood hearing loss. One-hundred and seventy-seven (65.8%) claimed to have poor knowledge in the areas included in the questionnaire. However, 232(86.2%) parents showed highly positive and favorable attitudes towards accessing pediatric audiology services, whereas 37(13.8%) yielded a negative attitude.

### 3.3. Association between Knowledge, Attitudes, and Socio-Demographic Characteristics

The association between knowledge, attitudes, and socio-demographic characteristics (Table 3) revealed a significant correlation between education levels and knowledge (*p* = 0.001). However, no significance was found between attitude factors and education levels. Further, there was a significant association between age group and knowledge level (*p* = 0.020).

The internal consistency of the questionnaire was assessed with the Cronbach’s alpha coefficient (α) and there was an excellent consistency for knowledge (0.86) and attitudes (0.90); an α closer to 1 is an indication of high reliability [16].

## 4. Discussion

The current study attempted to explore the knowledge and attitudes of parents towards childhood hearing loss and hearing services. The results from the study revealed that both fathers and mothers reported relatively poor knowledge of childhood hearing loss and its risk factors.

Around 58% of fathers reported a good degree of knowledge regarding consanguineous marriages causing hearing loss. More than half (59%) of mothers were aware that family history can cause hearing loss. These findings are consistent with the study reported by Kasper et al. [11]. Both parents have reported good knowledge regarding the statement “babies can be born with hearing loss.” Similar findings were reported in the study done in Saudi Arabia [9], whereas contradicting responses were reported from the Solomon Islands and India [11,12]. 

The current study also noted poor levels of knowledge for the risk factors of “high fever,” “measles”, “and “neonatal jaundice,” and the lowest knowledge level regarding “maternal rubella can cause hearing loss.” Both parents reported that they were unsure and had limited knowledge regarding whether drugs or medication cause hearing loss. This concurred with the study reported from Saudi Arabia [9] and India [12]. In addition, poor degree of knowledge regarding “low birth weight and hearing loss” was seen in our study, similar to the responses obtained from the regional data [9].

When probed about their knowledge level of OM and its risk factors, fathers (55%) reported a higher level of knowledge than mothers. However, this contrasted with findings from other regions where mothers were more aware of OM leading to hearing loss [17]. Several other studies report that both parents have an equal level of knowledge about OM and hearing loss [9,18]. Uncertainty was noted among most parents regarding “breast-feeding for first 6 months reduces or prevents OM.” However, contradictory findings were seen in a study by Kasper et al. [11], where participants claimed to be familiar with the subject of breastfeeding and prevention of OM. 

Reported knowledge levels regarding identification and intervention of hearing loss revealed comparatively higher percentages than parental awareness of risk factors leading to hearing loss. The parents’ knowledge about children with hearing loss attending school was excellent, rating beyond 65%. This was consistent with previous study findings [9,12]. In addition, fathers demonstrated greater knowledge than mothers regarding hearing loss identification soon after birth. Anecdotally, such gaps in the awareness and knowledge levels among mothers could be attributed to the lack of community awareness programs among expectant mothers [19]. Both parents showed significantly more knowledge (over 65%) regarding speech and language problems indicative of hearing loss. Few studies have explored this parameter in the past, except Kasper et al. [11]. They reported poor knowledge levels among parents.

Regarding the parents’ attitude towards utilizing pediatric audiology services, most parents were overwhelmingly positive and were in favor of newborn hearing screening immediately after birth (fathers: 90.4% and mothers: 89.1%). Furthermore, parents displayed a positive attitude towards testing their child for hearing loss at school. These are interesting findings, given the fact that successful implementation of newborn hearing screening (NHS) and other hearing screening methods in school were highly dependent on parents’ acceptance [20,21,22]. Our findings are consistent with the regional study [9]. It was encouraging to see a positive attitude from parents towards pediatric audiology services. Hence, the need for rolling out awareness programs at the grass root level for parents is instrumental for the optimal implementation of both the NHS and school-level hearing screening programs.

The current study also compared the association between knowledge and attitudes between age groups, revealing that parents aged 31–40 years (53%) share greater knowledge levels compared to other age groups. However, contradictory findings were reported from Saudi Arabia where parents aged more than 40 years showed better knowledge levels than those below [9]. Furthermore, comparative analysis of the levels of parents’ education denoted that university graduates have a greater degree of knowledge and comprehension compared to those with a secondary level of schooling (fathers: 83.7% and mothers: 62.7%, respectively).

It was surprising to note that 86.2% of parents showed a positive and favorable attitude towards hearing screening. However, a vast majority of them (65.8%) noted poor knowledge and comprehension regarding hearing loss and the associated risk factors. These findings are the first to be reported in the UAE. It highlights the need for formulating robust public awareness campaigns among expectant parents regarding childhood hearing loss. 

### 4.1. Implications

The current study findings highlight the dire need for initiating community outreach programs on hearing loss in children amongst parents in the UAE. Given the dearth of information from the region, these findings will help in tailoring the health awareness and promotional activities for the community in the UAE. Maternal and antenatal classes for expectant parents may be utilized to educate parents on breastfeeding initiatives as a preventable cause of hearing loss and OM. Further they can be educated on early intervention with NHS and school hearing screening. Digital pamphlets and social media could be utilized to disseminate information on identifying childhood hearing loss early and its associated risk factors for expectant fathers and mothers.

### 4.2. Limitations

All participants were from the same hospital setting, where their child was treated. Selection bias does exist as the parents who were willing to take part in the study were approached. In addition, parents who had reading or writing difficulties opted out of the study. Further assessment of the current findings is warranted; other parents from the adjacent emirates in the region should be studied.

### 4.3. Future Directions

Future studies could focus on understanding the implementation of an NHS program and assess the knowledge and attitudes of mothers during follow-up visits. Furthermore, a larger cohort of parents is to be recruited within the adjacent emirates allowing us to better profile the gap in knowledge levels among parents pertaining to childhood hearing loss and its prevention. In addition, focused groups or interviews with the parents to explore their beliefs shall help in tailoring the awareness campaigns.

## 5. Conclusions

The current study provides data on parental knowledge and attitudes towards childhood hearing loss and pediatric audiology services in the UAE. The findings outline the gap present in the region regarding parental awareness. Health promotional activities and community awareness campaigns are necessary to reduce the knowledge gap and to enhance parental engagement. Such a systematic approach would allow for the optimal implementation of an NHS program and to increase uptake for pediatric audiology services in the region.

## Figures and Tables

**Table 1:** Knowledge and attitudes of the parents towards childhood hearing loss and pediatric hearing services.

Survey Statement	Father (*n* = 94)			Mother (*n* = 175)		
	Yes (%)	No (%)	Unsure (%)	Yes (%)	No (%)	Unsure (%)
Knowledge: SNHL * Risk Factors						
1. Babies can be born with HL *	48.2	30.6	21.2	44.6	33.1	22.3
2. High fever can cause HL	31.5	45.1	23.4	38.3	45.6	16.1
3. Measles can cause HL	17.6	44.3	38.1	8.6	51.1	40.3
4. Maternal rubella can cause HL	05.5	46.3	48.1	11.1	46.2	42.7
5. Drugs/medications can cause HL	24.4	41.4	34.2	23.8	38.1	38.1
6. Jaundice can cause HL	17.3	39.4	43.3	21.5	37.1	41.4
7. Delayed crying at birth can cause HL	32.6	31.1	36.3	22.5	51.4	26.1
8. Family history can cause HL	46.1	27.2	26.7	58.3	31.4	10.3
9. Noise exposure can cause HL	40.5	29.1	30.4	25.2	44.3	30.5
10. Consanguineous marriage can cause HL	57.5	17.4	25.1	42.4	26.4	31.2
11. Low birth weight < 1500 g can cause HL	14.5	32.1	53.4	7.3	39.5	53.2
Knowledge: OM * and CHL * Risk-Factors						
12. Ear discharge and OM can cause HL	55.1	30.3	14.6	47.2	31.1	21.7
13. Recurrent flu can cause OM	29.8	26.1	44.1	13.5	35.2	51.3
14. Breast-feeding for first 6months reduces/prevents OM	28.2	44.5	27.3	16.6	45.2	38.2
15. Smoke (tobacco/woodfire) can predispose to OM	34.1	35.2	30.7	21.5	46.3	32.2
16. Routine childhood immunizations can reduce OM	33.3	29.4	37.3	37.1	28.7	34.2
Knowledge:Identification and Intervention						
17. HL can be identified soon after birth	53.2	14.2	32.6	43.1	19.2	37.7
18. Speech/language problems can be a sign of HL	69.5	18.4	12.1	61.5	14.3	25.2
19. Treatment for HL is available	43.5	14.1	42.4	42.6	11.1	46.3
20. Children with HL can attend school	77.1	5.2	17.7	56.5	11.4	32.1
Attitudes towards Pediatric Audiology Services						
21. I would like my baby tested soon after birth	90.4	5.2	4.6	89.1	7.5	3.4
22. I would accept OAE */AABR * hearing screening test for my baby	90.1	6.1	3.8	90.6	6.3	3.1
23. I would like my child tested at school	82.5	12.2	5.3	87.1	7.5	5.4
24. I would let my child use hearing aids	86.1	2.2	11.7	90.2	3.6	6.2
25. I would accept ear surgery for my child	82.5	1.4	16.1	82.3	3.3	14.4
26. I would like more information	81.6	5.2	13.2	90.2	3.5	6.3

* SNHL: sensorineural hearing loss, * HL: hearing loss, * OM: otitis media, * CHL: conductive hearing loss,* OAE: oto-acoustic emission, * AABR: automated auditory Brainstem response.

**Table 2:** Overall parental level of knowledge and attitudes.

Level	N (%) N = 269
Knowledge	
Good	92 (34.2)
Poor	177 (65.8)
Attitude	
Positive	232 (86.2)
Negative	37 (13.8)

**Table 3:** Association between knowledge, attitudes, and socio-demographic characteristics of parents.

Factor	Knowledge		Attitude	
	Good (*n* = 92) N (%)	Poor (*n* = 177) N (%)	Positive (*n* = 232) N (%)	Negative (*n* = 37) N (%)
**Age Group**				
21–30	39 (42.4)	99 (55.9)	117 (50.5)	21 (56.8)
31–40	49 (53.3)	66 (37.3)	102 (43.9)	13 (35.1)
41–50	4 (4.3)	12 (6.8)	13 (5.6)	3 (8.1)
*p*-value	0.020 *		0.549	
**Gender**				
Father	36 (39.1)	58(32.8)	79 (34.1)	15 (40.5)
Mother	56 (60.9)	119 (67.2)	153 (65.9)	22 (59.5)
*p*-value	0.299		0.442	
**Education Level**				
Secondary school or below	15 (16.3)	66 (37.3)	64 (27.6)	17 (45.9)
University	77 (83.7)	111 (62.7)	168 (72.4)	20 (54.1)
*p*-value	0.001 *		0.242	

* Significant at *p* ≤ 0.05 level.

## Data Availability

The data presented in this study are available on request from the corresponding author.
